# Estrogen receptor 1 (ESR1) regulates VEGFA in adipose tissue

**DOI:** 10.1038/s41598-017-16686-7

**Published:** 2017-12-01

**Authors:** L. A. Fatima, R. S. Campello, R. de Souza Santos, H. S. Freitas, A. P Frank, U. F. Machado, D. J. Clegg

**Affiliations:** 1 Department of Physiology and Biophysics, Institute of Biomedical Sciences, University of São Paulo, São Paulo, Brazil; 2 Biomedical Research Department, Diabetes and Obesity Research Division, Cedars-Sinai Medical Center, Los Angeles, California USA

## Abstract

Vascular endothelial growth factor A (VEGFA) is a key factor in the regulation of angiogenesis in adipose tissue. Poor vascularization during adipose tissue proliferation causes fibrosis and local inflammation, and is associated with insulin resistance. It is known that 17-beta estradiol (E2) regulates adipose tissue function and VEGFA expression in other tissues; however, the ability of E2 to regulate VEGFA in adipose tissue is currently unknown. In this study, we showed that, in 3T3-L1 cells, E2 and the estrogen receptor 1 (ESR1) agonist PPT induced VEGFA expression, while ESR1 antagonist (MPP), and selective knockdown of ESR1 using siRNA decreased VEGFA and prevented the ability of E2 to modulate its expression. Additionally, we found that E2 and PPT induced the binding of hypoxia inducible factor 1 alpha subunit (HIF1A) in the VEGFA gene promoter. We further found that VEGFA expression was lower in inguinal and gonadal white adipose tissues of ESR1 total body knockout female mice compared to wild type mice. In conclusion, our data provide evidence of an important role for E2/ESR1 in modulating adipose tissue VEGFA, which is potentially important to enhance angiogenesis, reduce inflammation and improve adipose tissue function.

## Introduction

Adipose tissue is a highly vascularized tissue and there are data to support ‘healthy’ expansion of adipose tissue occurs when adipose tissue is fully vascularized^1,2^. Importantly, ‘unhealthy’ expansion of adipose tissue, which occurs in obesity, is associated with relatively nonvascularized adipose tissue leading to inflammation, ectopic fat deposition, and insulin resistance^3,4^. Vascular Endothelial Growth Factor A (VEGFA) is an angiogenic factor in adipose tissue^5^; however, the regulation of VEGFA in adipose tissue remains unclear. Interesrtingly, while VEGFA is important for the development of new vessels it is also considered a pro-inflammatory cytokine^2,6^, potentially indicating a differential effect of VEGFA. When VEGFA is overexpressed in adipose tissue, it results in increased blood vessel number and size, protection against high-fat diet–induced hypoxia and obesity, and improves whole-body insulin sensitivity and glucose tolerance^2^. On the other hand, there are data to suggest that blocking neovascularization in adipose tissue prevents the development of obesity^7,8^, further suggesting that the impact of VEGFA on adipose tissue function has yet to be fully understood.

17 beta-estradiol (E2) is a hormone that is critically involved in reproduction as well as energy homeostasis^9,10^. E2 has been demonstrated to regulate VEGFA in a variety of tissues^11^, but it is unclear if E2 modulates VEGFA in adipose tissue. Within adipose tissue, E2 can suppress lipid accumulation, adipocyte size, fatty acid uptake, lipogenesis, inflammation and fibrosis^12–14^. Reductions in circulating E2, as seen in ovariectomized mice and rats, induce insulin resistance and increase susceptibility to high-fat diet induced-metabolic disorders^15^. Moreover, E2 is hypothesized to be a key feature in the sexual dimorphisms observed in body fat accumulation and adipose tissue expandability. Males accumulate visceral fat, which has been highly correlated to increased cardiovascular risk and tends to be more fibrotic and less vascularized; while females accumulate fat in the subcutaneous depot prior to menopause, and subcutaneous adipose tissue is highly vascularized and less fibrotic. It is further predicted that subcutaneous accumulation of adipose tissue is one of the features that protects pre-menopausal women from diseases associated with obesity and the metabolic syndrome^16,17^.

E2 binds to its classical receptors- estrogen receptor 1 (ESR1) and estrogen receptor 2 (ESR2) and triggers genomic and non-genomic actions^18–20^. E2 can also bind to a membrane-bound G-protein-coupled estrogen receptor (GPER)^21^. The expression and function of estrogen receptors (ERs) are tissue specific^18,20,22,23^. The classical mechanisms of E2/ERs interactions include ligand binding, receptor dimerization and activation of estrogen response elements (ERES) in target genes leading to activation or repression of genes^24,25^. In addition, E2/ERs can act rapidly via extracellular and membrane located receptors^26^. Furthermore, E2 can modulate gene expression by interacting with its receptors, which in turn activate other transcription factors that bind to DNA^26,27^. As an example, hypoxia-inducible factor 1 alpha subunit (HIF1A) is a transcription factor proposed as a candidate for E2-induced regulation of the *VEGFA* gene in non-adipose tissues^28^.

Human subcutaneous and visceral adipose tissue expresses both receptors, ESR1 and ESR2^29,30^. In 3T3-L1 cells and in mouse adipocytes, ESR1 is predominant, but both isoforms are co-expressed in the nucleus of adipocytes^30–32^. ESR1 is thought to be the predominate ER involved in the regulation of adipose tissue function^13^. Total body deletion of ESR1 as well as tissue-specific knockdown of ESR1 in male and female mice promotes increased adiposity, fibrosis, insulin resistance and glucose intolerance^33–35^. On the other hand, total body ESR2 knockout mice do not present with alterations in body composition and glucose homeostasis^36,37^, suggesting a more predominate role for ESR1 in regulating adipose tissue function, yet the mechanisms by which it does so are currently unknown.

As previously mentioned, there are data to suggest that the VEGFA gene is transcriptionally regulated by direct E2-dependent ESR1 binding and activation^38,39^; however, it is not known if this regulation occurs in adipose tissue. Therefore, we hypothesized that VEGFA gene is directly regulated by E2-dependent ESR1 binding and activation in adipose tissue, which would in turn improve adipose tissue angiogenesis and promote metabolically healthy adipose tissue. To probe for the involvement of E2/ESR1 in the activation of VEGFA in adipose tissue, we explored the mechanisms by which E2 interacts with ESR1 to regulate VEGFA.

## Results

### E2 induces VEGFA mRNA and protein in 3T3-L1 cells

**Figure 1:**
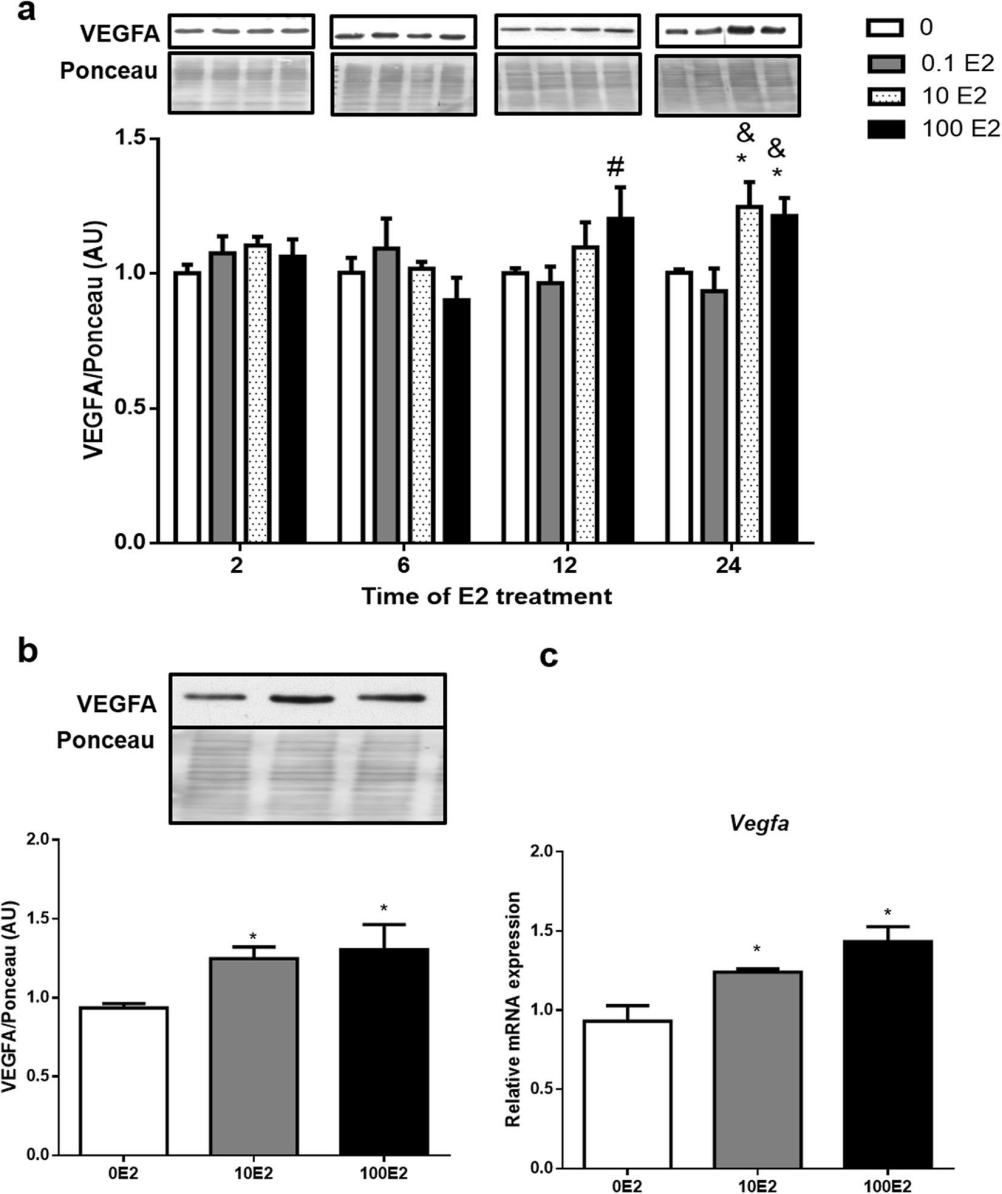
E2 effects on VEGFA mRNA and protein expression: 3T3-L1 adipocytes were treated for 2, 6, 12 and 24 hours with 0.1, 10 nM and 100 nM of E2. Cells treated only with vehicle were used as control (0E2). VEGFA protein (**a** and **b**) and mRNA (**c**) were analyzed by Western blotting and qPCR, respectively. In panel b, are shown the graph with the results referent to the treatments with 10 and 100nM of E2 for 24 hours with a representative autoradiogram. Full-length blots of the cropped image are presented in Supplementary Figure S1. Data on the graphs represent mean ± SEM of 5 to 8 samples. ^*^P < 0.05 *vs* 0E2, ^#^P < 0.05 vs 0.1E2, ^&^P < 0.05 vs 0.1 E2 in the same time.

We first investigated the impact of E2 across a time-course on VEGFA protein, and to do so we used fully differentiated 3T3-L1 adipocytes which were treated with 0.1, 10 and 100 nM of E2 for 2, 6, 12 and 24 hours. We found that treatments with 10 and 100 nM of E2 for 24 hours were capable of increasing VEGFA protein when compared to control treated cells (Fig. 1a and b). Consistently we also found that *Vegfa* mRNA was increased by 10 and 100 nM E2 within 24h when compared to control treated cells (Fig. 1c). As both doses of E2 (10 and 100 nM) were effective at increasing VEGFA gene expression and protein, the following experiments were performed using the lower dose, 10 nM for 24 hours, which is a physiological concentration of E2 *in vivo* ^40^.

### E2 treatment increases ESR1 mRNA expression in 3T3-L1 cells

**Figure 2:**
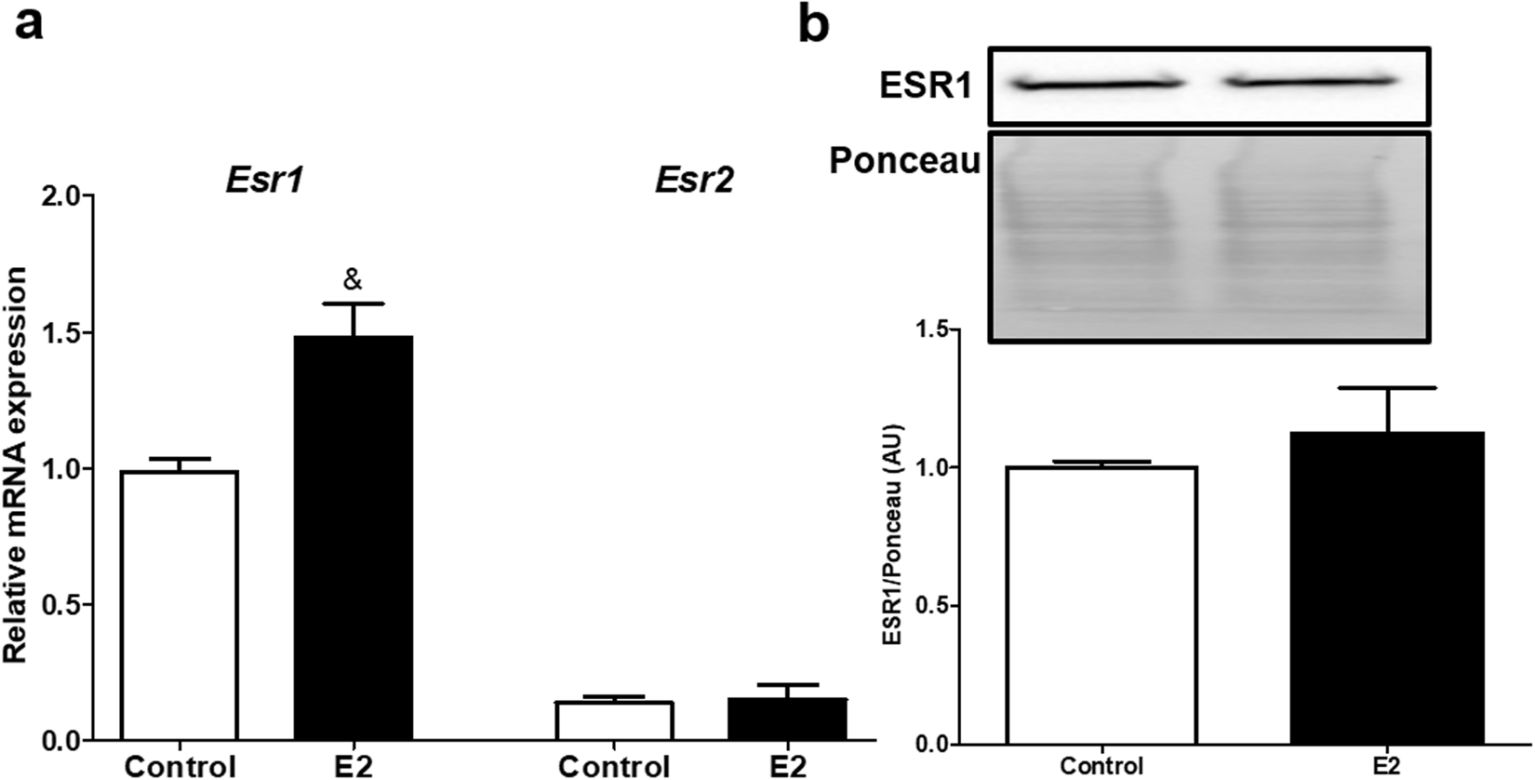
(**a**) mRNA expression of *Esr1* and *Esr2* after treatment with 10 nM of E2 for 24 hours in 3T3-L1 cells. (**b**) ESR1 total protein expression after treatment with 10 nM of E2 for 24 hours in 3T3-L1 cells. Full-length blots of the cropped image are presented in Supplementary Figure S1. Control cells were treated with vehicle (water). Data on the graphs are mean ± SEM of 3 to 5 samples. ^&^P < 0.05 *vs* control.

The mRNA expression of *Esr1* and *Esr2* was analyzed after treatment with 10 nM of E2 for 24 hours. E2 increased *Esr1* expression, but did not change ESR1 protein (Fig. 2a and b), while *Esr2* mRNA expression was not affected by the E2 treatment. Interestingly, we determined that 3T3-L1 cells have more *Esr1* than *Esr2* transcripts (Fig. 2a).

### ESR1 agonist PPT enhances VEGFA expression while ESR2 agonist DPN represses it in 3T3-L1 cells

**Figure 3:**
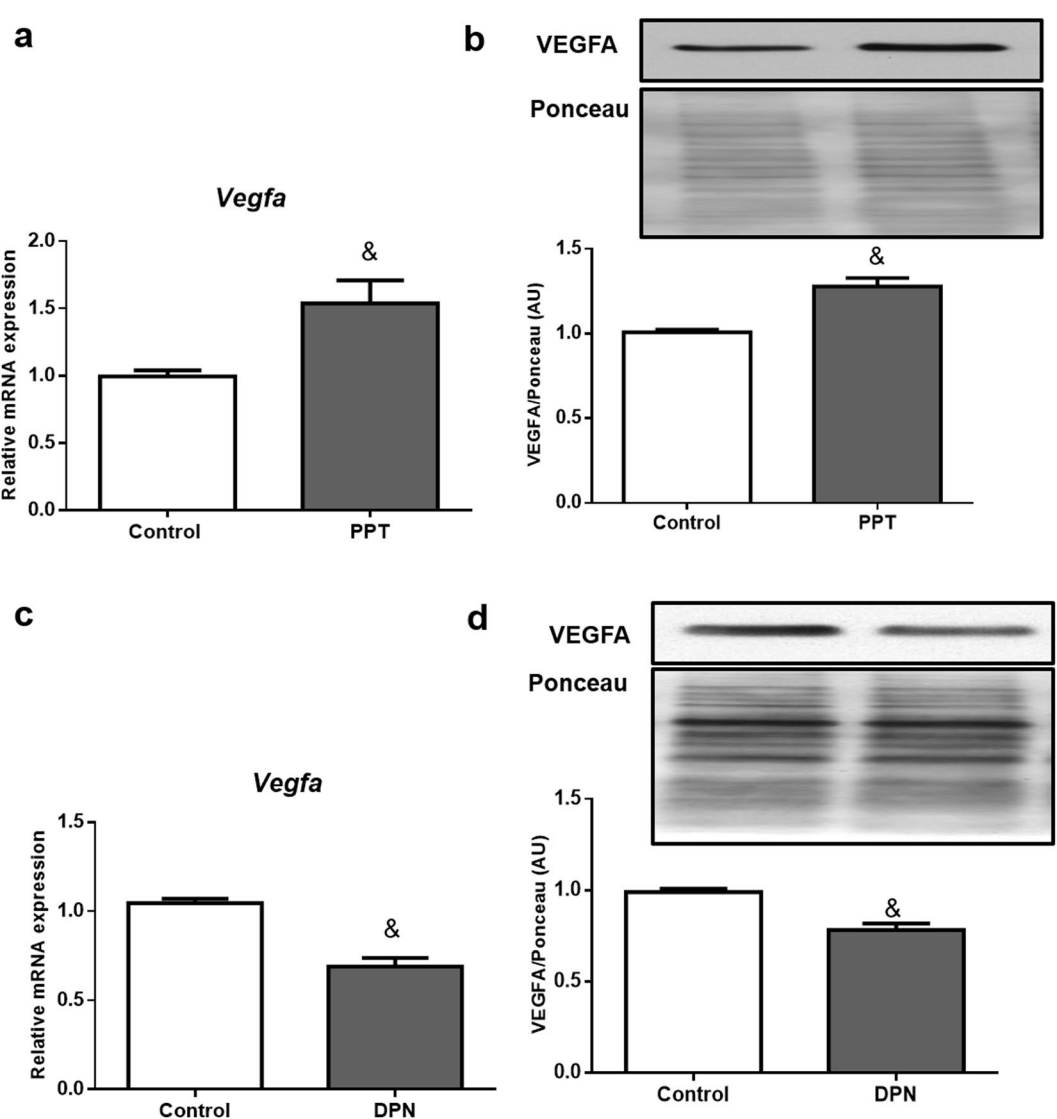
Effects of ESR1 agonist (PPT) and ESR2 agonist (DPN) on VEGFA mRNA and protein: 3T3-L1 adipocytes were treated for 24 hours with 10 nM of PPT (**a**,**b**) or 10 µmol/L of DPN (**c**,**d**). Full-length blots of these cropped images are presented in Supplementary Figure S1. Cells treated with vehicle (0.1% DMSO) were used as control. The representative autoradiograms are shown on top. Data are shown as the mean ± SEM of 6 samples. ^&^P < 0.05 vs control.

To determine the role of ERs to regulate VEGFA, we first probed the activity of ESR1 and ESR2 using their agonists. ESR1 agonist PPT, independently of E2, induced VEGFA mRNA and protein expressions (Fig. 3a and b). Interestingly, when ESR2 agonist DPN was used, VEGFA mRNA and protein were decreased (Fig. 3c and d), demonstrating an opposite effect of ERs on the regulation of VEGFA in adipocytes.

**Figure 4:**
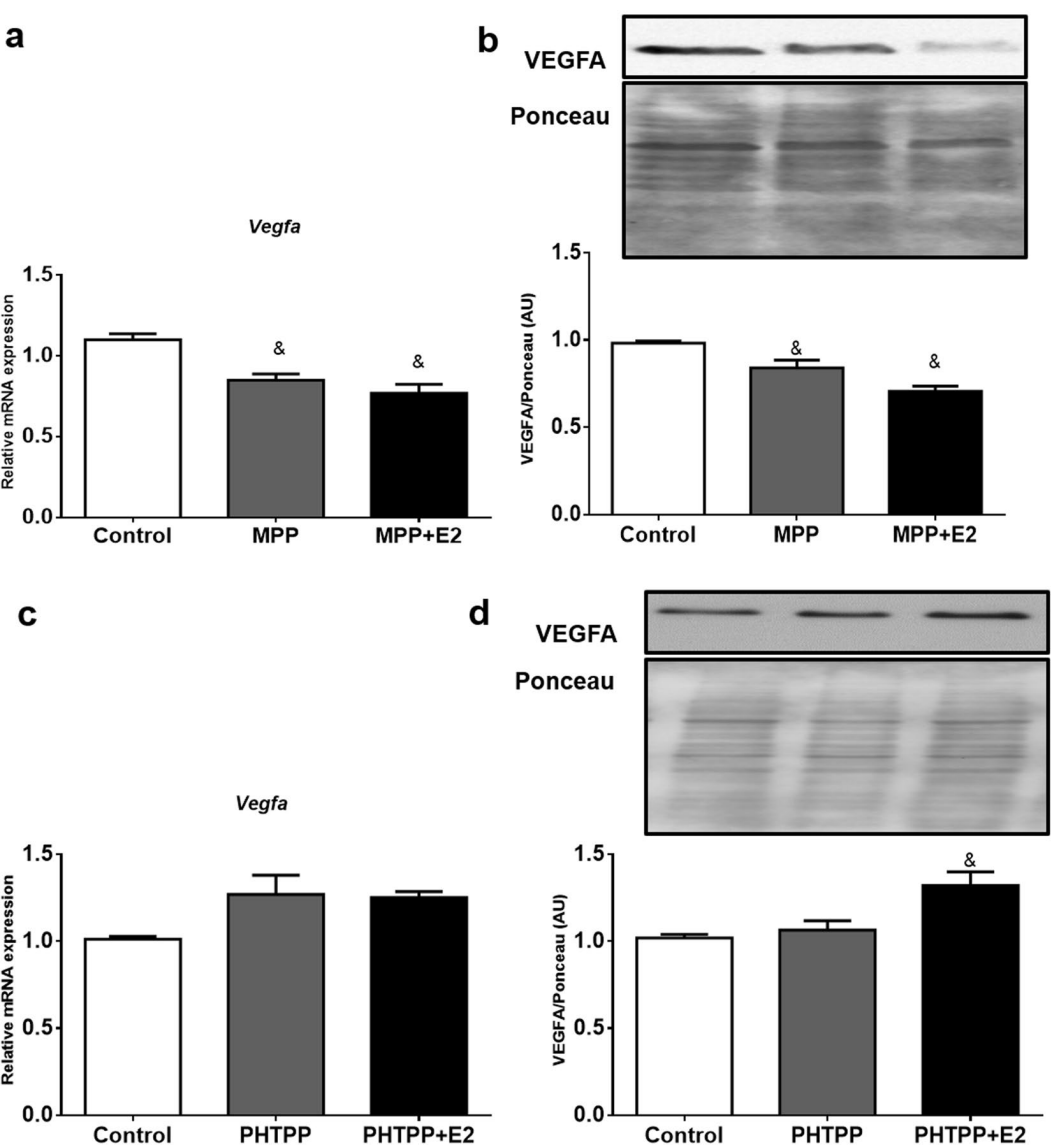
Effects of ESR1 antagonist (MPP) and ESR2 antagonist (PHTPP) on VEGFA mRNA and protein: 3T3-L1 adipocytes were treated for 24 hours with 10 µmol/L of MPP (**a**,**b**) or 100 nmol/L of PHTPP (**c**,**d**), in the absence or presence of 10 nM of E2. Full-length blots of these cropped images are presented in Supplementary Figure S1. Cells treated with vehicle (0.1% DMSO) were used as control. The representative autoradiograms are shown on top. Data are shown as the mean ± SEM of 6 samples. ^&^P < 0.05 *vs* control.

**Figure 5:**
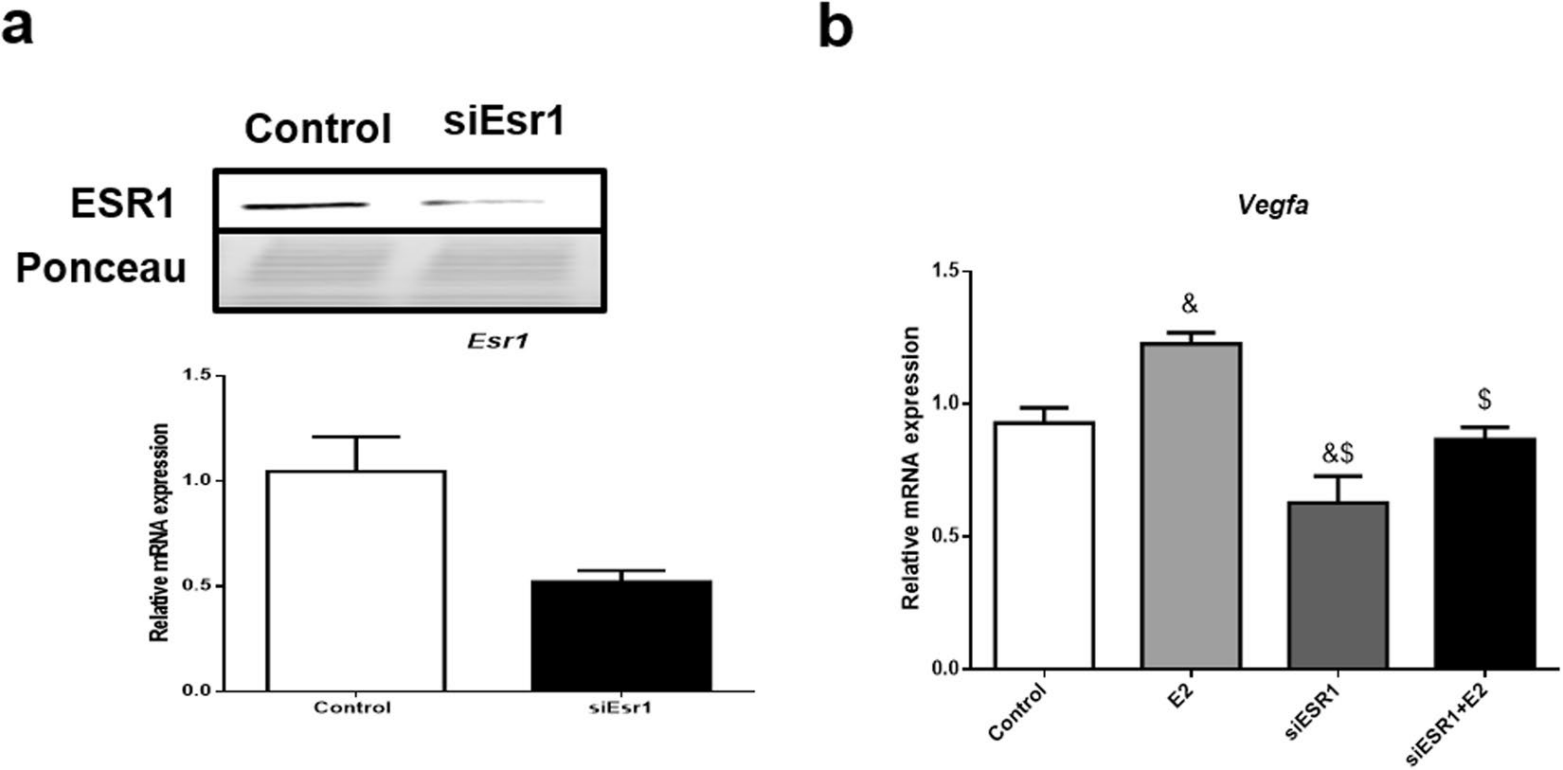
Effects of *Esr1* silencing on VEGFA mRNA expression. *Esr1* gene was knocked-down using siRNA, after 48 hours cells were treated with 10 nM of E2 and collected after 24 hours. Data were presented in mean ± SEM values. ^&^p < 0.05 *vs* control; ^$^p < 0.05 *vs* E2 (n = 3 independent rounds of cells).

To test whether blocking ESR1 activity specifically reduces VEGFA expression in adipocytes, we used the ESR1 antagonist MPP and found that it decreased VEGFA mRNA and protein and prevented the ability of E2 to induce VEGFA expression (Figs 4a and b). On the other hand, the ESR2 antagonist PHTPP did not affect VEGFA, but in the presence of 10 nM of E2, VEGFA protein was increased (Fig. 4c and d). Additionally, we used an siRNA for ESR1 and further demonstrated a reduction in ESR1 mRNA and protein, as well as a reduction in VEFGA mRNA (Fig. 5a,b). Also, we demonstrate following reductions in ESR1 with the siRNA, adding back E2 does not restore VEGFA expression (Fig. 5b).

### E2 induces the binding of HIF1A in the VEGFA promoter in 3T3-L1 cells

**Figure 6:**
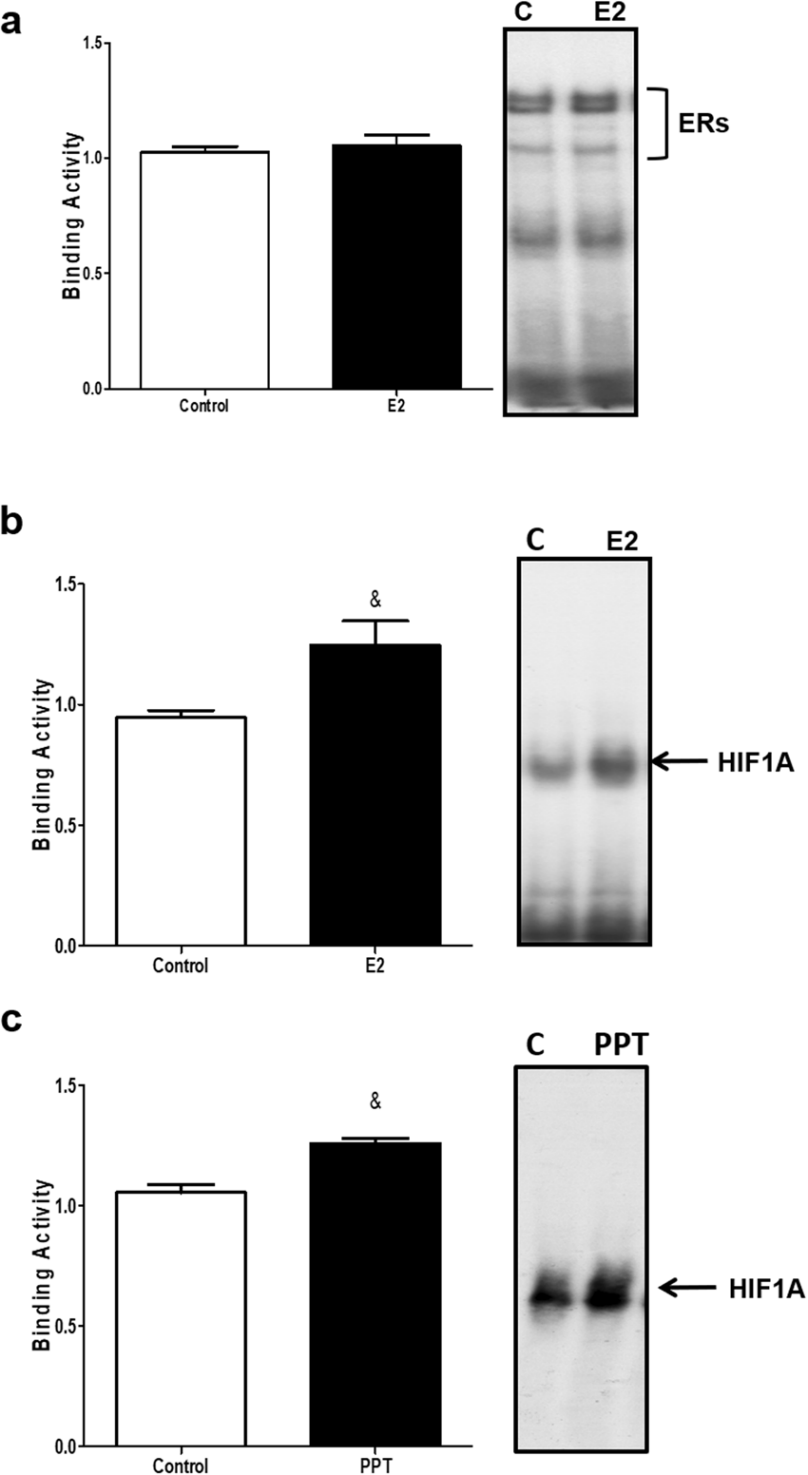
Effect of E2 on ERs and HIF1A binding activity to the *Vegfa* gene promoter. 3T3-L1 adipocytes were treated with vehicle (0.01% DMSO; Control); 10 nM of E2 or 10 nmol/L of PPT for 24 hours. EMSA was performed as described in Materials and Methods. (**a**) ERs, biding in VEGFA promoter after 24 hours of E2 treatment. (**b**) HIF1A biding in VEGFA promoter after 24 hours of E2 treatment; (**c**) HIF1A binding in VEGFA gene promoter after 24 hours of PPT treatment. Representative images of the experiments are in the right. Results represent three independent experiments. Data are shown as the mean ± SEM of 4 to 6 samples. ^&^P < 0.05 *vs* control.

First, we tested if treatment with E2 induced direct binding of ERs in the ERE sites in the VEGFA promoter, using an Electrophoretic mobility shift assay (EMSA). We observed two DNA/protein complexes corresponding to the binding of ERs in the VEGFA promoter and both receptors, ESR1 and ESR2, are present in these complexes, suggesting ESR1 and ESR2 directly interact with the VEGFA gene promoter in adipocytes (Figure S2). Furthermore, we did not observe an enhancement of the effect following E2 exposure in the intensity of the complex 1 and 2 (Fig. 6a), suggesting that the treatment with E2 did not increase the direct binding of ERs in the VEGFA gene.

We next tested whether HIF1A may be involved in VEGFA upregulation by E2. To do this, we analyzed the binding of HIF1A to the VEGFA promoter (Figure S3). Treatment with 10 nM of E2 for 24 hours increased the DNA–protein complex signal when compared to the control cells (Fig. 6b). In addition, treatment with the ESR1 agonist PPT also increased the DNA-protein complex intensity (Fig. 6c). These results reveal that HIF1A participates in the regulation of VEGFA mediated by E2/ESR1 in adipocytes.

### The lack of ESR1 reduces VEGFA expression in different fat depots of female mice

To further explore the interaction between ESR1 and VEGFA expression *in vivo*, we investigated their expression in inguinal (iWAT) and gonadal (gWAT) adipose tissues from wild-type (WT) and total body ESR1 knockout (ERKO) mice.

**Figure 7:**
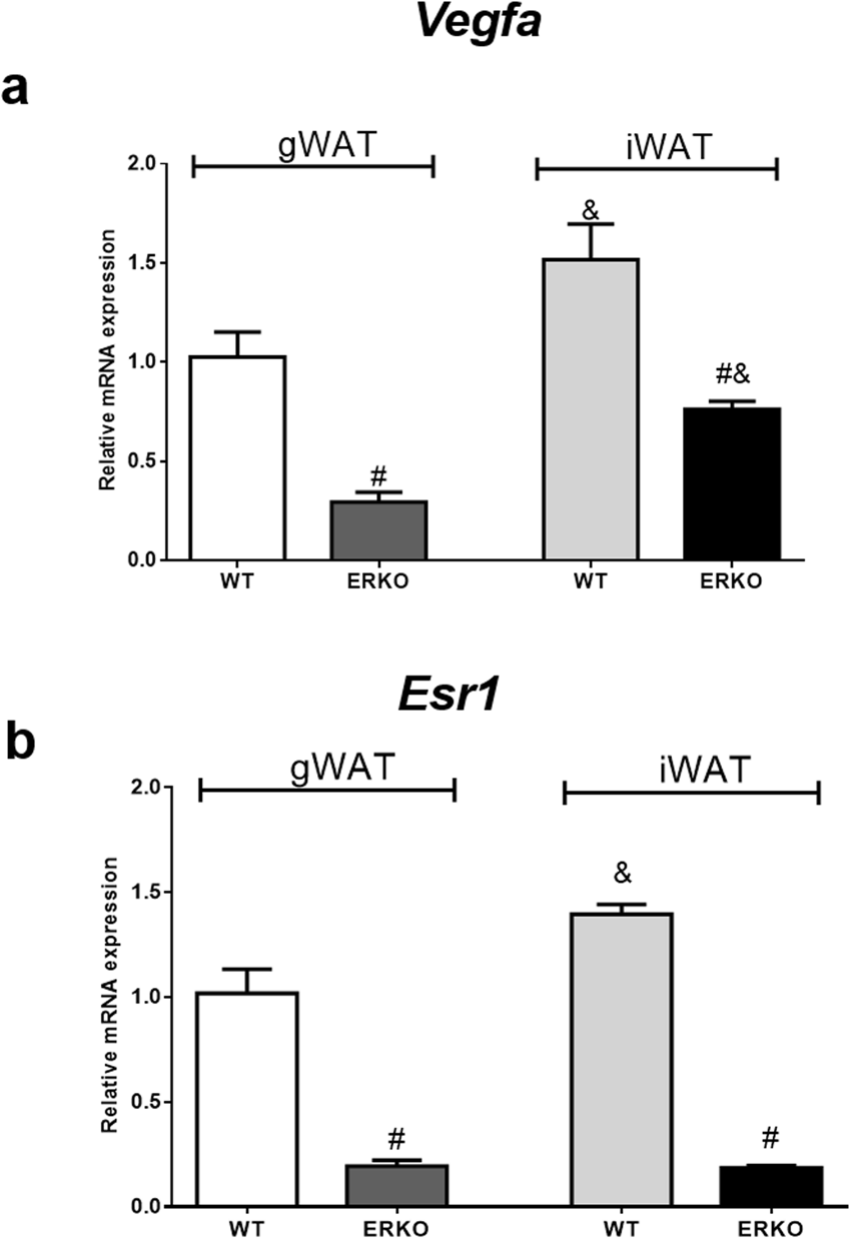
*Vegfa* (**a**) and *Esr1* (**b**) mRNA expression in gonadal (gWAT) and in inguinal (iWAT) adipose tissues from WT and ERKO female mice. Data on the graphs are means ± SEM of 4–7 samples, ^#^p < 0.05 *vs* WT; ^&^p < 0.05 *vs* gWAT WT.

**Figure 8:**
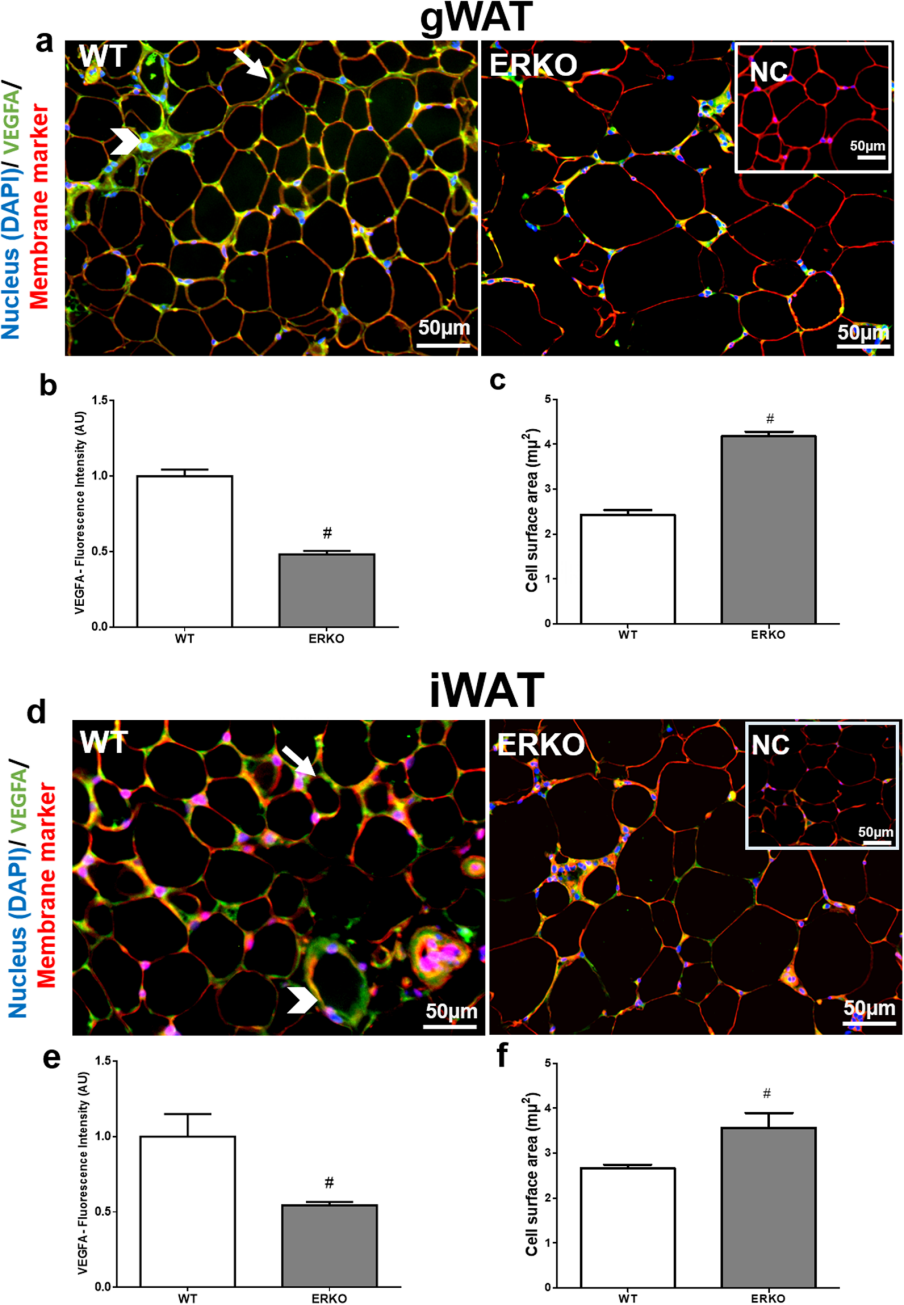
VEGFA protein expression in gonadal (gWAT) and inguinal (iWAT) adipose tissue from WT and ERKO female mice. Immunofluorescence staining of VEGFA in gWAT (**a**) and iWAT (**d**). Nucleus in blue (DAPI), VEGFA in green, membrane marker in red, and composite in yellow/orange; arrows indicate VEGFA expression in adipocytes and arrowheads in vessels; scale 50 μm; Fluorescence intensity quantification in gWAT (**b**) iWAT (**e**). Adipocyte size (surface area- μm^2^) in gWAT (**c**) iWAT (**f**). Data on the graphs are means ± SEM of 4–7 samples, ^#^p < 0.05 *vs* WT. NC = negative control.

*Vegfa* mRNA expression was lower in the iWAT and gWAT adipose tissues of ERKO compared to WT mice (Fig. 7a). Moreover, iWAT adipose tissue has more *Vegfa* and *Esr1* mRNA expression than gWAT (Fig. 7a and b). We also analyzed the protein by immunofluorescence and quantified fluorescence intensity. In the images, VEGFA is stained green and membrane marker is stained red. VEGFA-positive staining is observed in vessels and adipocytes (Fig. 8a and d). The intensity of VEGFA-positive staining was reduced in iWAT and gWAT adipose tissues from ERKO compared to WT mice (Fig. 8a,b,d and e). Thus, it appears that ESR1 is important in the regulation of VEGFA in both adipose tissue depots.

In addition, measurement of adipocyte size was performed to demonstrate the role of ESR1 in adipose tissue morphology. Adipocyte hypertrophy has been demonstrated to impair adipose tissue function by inducing local inflammation, mechanical stress, alterations in metabolism, and insulin resistance^41^. Consistent with previous findings, adipocytes from ERKO were larger than those from WT mice in iWAT and gWAT (Fig. 8c and f) further supporting the notion that lacking ESR1 induces adipocyte hypertrophy.

**Figure 9:**
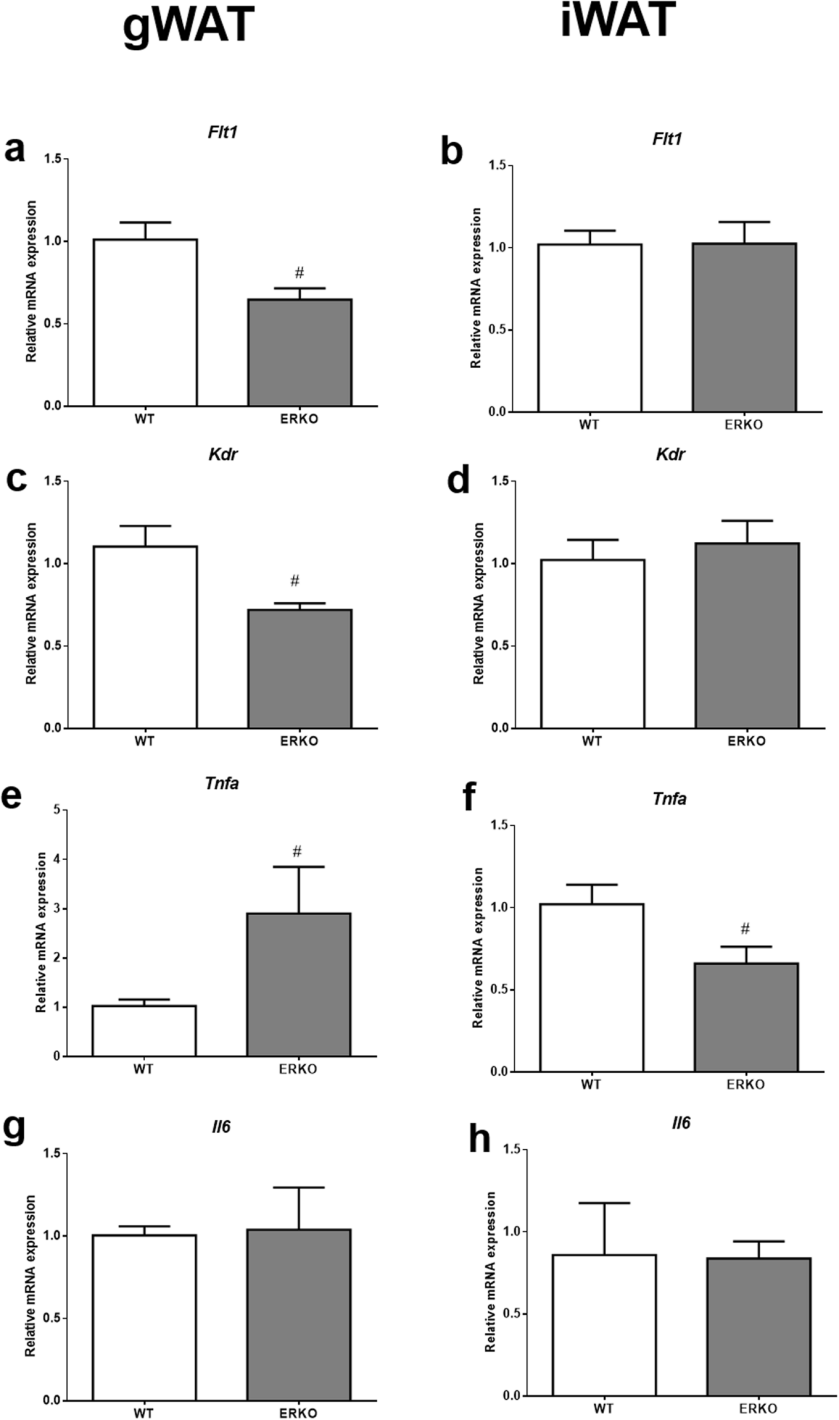
Relative mRNA expression of *Vegf* mRNA receptors, and inflammatory markers in iWAT and gWAT from WT and ERKO female mice. (**a** and **b**) *Flt1* in gWAT and iWAT respectively; (**c** and **d**) *Kdr* in gWAT and iWAT respectively; (**e** and **f**) *Tnfa* in gWAT and iWAT respectively; (**g** and **h**) *Il6* in gWAT and iWAT respectively. Data on the graphs are means ± SEM of 3–7 samples, ^#^p < 0.05 *vs* WT.

To further explore the role of ESR1 and its impact on adipose tissue function, mRNA expression of genes related to VEGFA signaling and markers of inflammation were analyzed. VEGFA binds to and activates VEGFR1 (Flt1) and VEGFR2 (KDR/Flk1). The major pro-angiogenic signal is generated from the ligand-activated KDR, and KDR can be considered a vascular marker. In iWAT, we found neither *Flt1* nor *Kdr* mRNA expression was significantly different in ERKO when compared to WT mice (Fig. 9b and d). However, both *Flt1* and *Kdr* genes were downregulated in gWAT of ERKO mice (Fig. 9a and c).

We further found that in gWAT, the proinflammatory marker, tumor necrosis factor alpha (*Tnfa*) mRNA expression was higher in ERKO when compared to WT mice (Fig. 9e); however, in iWAT, *Tnfa* mRNA expression was lower in ERKO mice compared to WT mice (Fig. 9f). It is important to highlight that the expressions of *Tnfa* and VEGFA were opposite in the gWAT. No significant differences were observed for *Il-6* expression in either iWAT or gWAT (Fig. 9g and h).

## Discussion

Reductions in circulating E2 levels are associated with weight gain, increased visceral adiposity, an increased prevalence of obesity, insulin resistance and type 2 Diabetes^12,42^. It is known that E2 deficiency leads to increased expression and secretion of proinflammatory cytokines^12,43^. There are data to suggest that VEGFA is a proinflammatory cytokine, and in some tissues, that VEGFA is regulated transcriptionally by E2^11^, but what is not known is the interaction between E2 and VEGFA in adipose tissue. VEGFA is also an important angiogenic factor in adipose tissue and regulates the vascular infrastructure required for healthy expansion of adipose tissue^1,2,5^. In addition, angiogenesis plays a crucial role in the regulation of adipogenesis^44^. Here we demonstrate VEGFA is regulated by E2 in 3T3-L1 cells and adipose tissues, and this regulation appears to be through its interaction with ESR1.

In 3T3-L1 cells exposed to E2, both ESR1 and VEGFA were upregulated compared to control cells, and the activation of ESR1 *per se* by PPT also increased VEGFA expression. On the other hand, the inhibition of ESR1 or the activation of ESR2 decreased VEGFA expression. These data suggest, that the ERs regulate VEGFA in an opposite way. It is known that the decline of E2 levels in postmenopausal women is associated with reductions of ESR1 expression, as well as there is a change in ESR1/ESR2 ratio such that there is more ESR2 relative to ESR1^45^. In addition, the postmenopausal period is characterized by increased visceral adiposity and enhanced metabolic risks associated with obesity^46^, which may reflect either the reductions in E2 and/or ESR1 expression, and their relative impact on VEGFA.

Previously, it has been demonstrated that VEGFA is regulated positively by both ERs^11^. In addition, there are data to suggest that in cancer cells, there is an inhibition of VEGFA following treatment with E2 that occurs when both ERs are present, but there is an induction of VEGFA when ERs are expressed alone^47^. E2 has also been shown to stimulate angiogenesis primarily via ESR1, but again this was not known for adipose tissues^48^. In our animal model, there was a lower expression of VEGFA in ERKO compared to WT mice in gWAT and iWAT. In addition, we demonstrated a positive correlation between the amount of ESR1 and VEGFA, which differs by adipose tissue depots. Specifically, we found that iWAT expressed more ESR1 and VEGFA mRNA than gWAT. These findings indicate that ESR1 might play an important role in the regulation of VEGFA in white adipose tissues. It has been shown that in obese humans, VEGFA expression and angiogenic potential are similar between subcutaneous and visceral adipose tissues^49^. Moreover, others showed that in individuals with abdominal obesity, stimulation of subcutaneous adipose tissue angiogenesis promoted adipocytes hyperplasia, thus enhancing the storage capability of adipose tissue, which, in turn, is a protective effect against metabolic disorders^3^.

Our data further suggests that E2 does not regulate VEGFA in 3T3-L1 cells via direct binding of ERs to the VEGFA gene, which is not consistent with other cells^11^. However, there are data demonstrating that E2 is also able to augment gene expression through its interaction with ERs, which may in turn bind to other transcriptional factors^23^, such as HIF1A, to regulate VEGFA^48,50,51^. Adipose tissue HIF1A activity is influenced by multiple signals, including adipogenesis, insulin, hypoxia, and obesity, but the induction of HIF1A by E2 can induce VEGFA and thereafter promote angiogenesis, a process that is required for adipocyte differentiation and adipose tissue growth^52,53^. HIF1A is the mediator of physiological and pathophysiological responses to hypoxia^54^; however, many studies have demonstrated that HIF1A regulates VEGFA expression in various cell types also in situations of normoxia^28^. There are data to suggest that HIF1A is a direct transcriptional target of ESR1 and that the HIF1A gene bears a canonical ER-binding element that responds to estrogen signaling, and these data demonstrate a direct regulatory link between the ESR1 and HIF1A pathways, but this has been demonstrated in breast cancer^55^. HIF1A is a labile protein that is rapidly degraded by many different factors, and we have previously reported that indeed E2/ESR1 transcriptionally regulates PHD3 which targets HIF1A for ubiquination and degradation within adipose tissue^56^. We speculate that E2/ESR1 binds to the ER-binding element within the HIF1A gene to facilitate angiogenesis by then inducing VEGFA in conditions of normoxia; whereas, in conditions of hypoxia, E2/ESR1 binds to the promotor of PHD3 and targets HIF1A for degradation.

Here we observed that E2 through ESR1 activation induced HIF1A binding on the VEGFA promoter. However, the regulation regarding HIF1A in adipose tissue is controversial. It has been demonstrated that in adipose tissue HIF1A promotes fibrosis and inflammation, and E2/ESR1 can negatively regulate HIF1A transcription and degradation^56^. On the other hand, inhibition of HIF1A in adipose tissue induces obesity and glucose intolerance, due to the impairment of energy expenditure in mice^57^. Moreover, in the uterus, it has been demonstrated that E2 induced VEGFA expression via membrane ER and activation of PI3K/Akt pathway, which is required for the recruitment of HIF1 to the VEGFA gene promoter^28^. Indeed, our group has previously observed that treatments with E2 and ER-agonist PPT induced AKT phosphorylation in adipocytes^58^. Together, these data support the notion that E2/ESR1 can activate HIF1A in adipocytes to induce VEGFA expression. Acute induction of HIF1A binding to VEGFA can increase the vascularization in adipose tissue that in turn prevents the chronic hypoxia that is directly linked to metabolic dysfunction in adipose tissue^59^.

VEGFA binds to and activates its receptors, Flt1 and KDR. The major pro-angiogenic signal is generated from the ligand-activated KDR that is also considered a marker of vascular density^60^. The inhibition of VEGFA, which is induced by the inactivation of KDR, occurs during the early phase of high fat diet exposure and leads to weight gain and systemic insulin resistance^2^. Our findings indicate that *Kdr* mRNA expression was lower in gWAT from ERKO mice, which could impair VEGFA signaling, leading to reduced formation of new vessels and increased inflammatory markers, as demonstrated by a higher *Tnf* expression in gWAT of ERKO mice. There is a depot difference in the effect of ESR1 to regulate VEGFA receptors and inflammatory markers, and this could be due to the depot difference in the amount of ESR1. We observed that gWAT has less ESR1 mRNA expression than in iWAT and the gonadal depot was the most affected by inflammation. The health expandability of white adipose tissue has been linked to sexual dimorphisms of adipose tissues and gonadal adipose from female rats is reported to have a higher capacity to expand and has a lower level of inflammation when compared to male adipose tissue. Our data further suggest this may be due to the higher presence of E2 and ESR1 in female gonadal adipose tissue^13,17^.

VEGFA triggers angiogenesis in adipose tissue, as reflected by enhanced vascular density and improvements in systemic metabolism^61^. Moreover, it has been demonstrated that VEGFA has a role in the activation and expansion of BAT and “brown fat-like” adipocytes in the subcutaneous adipose tissue a process termed ‘beiging’ and this has been linked to improvements in energy homeostasis and of systemic metabolism^2,62^. We observed in BAT, *Vegfa* and *Kdr* mRNA expression was downregulated in ERKO mice compared to WT (Figure S4), suggesting that E2 may facilitate activation of BAT or beiging of white adipose tissue through ESR1/VEGFA induction.

Our data also demonstrated that visceral and subcutaneous adipocytes from ERKO had a larger cell surface area when compared to fat cells from WT mice. These results indicate that total body loss of ESR1 induces adipocyte hypertrophy, in agreement with results previously published^33,63^. Adipocyte hypertrophy is linked to inflammation and insulin resistance. Indeed, the larger adipocyte size was associated with a decrease in VEGFA expression, perhaps suggesting a reduction in vascularization in those tissues, and further supporting an important role for ESR1 in mediating vascularity of adipose tissue through increasing VEGFA to facilitate adipose tissue expansion^7^. Finally, there are data to suggest that ablation of VEGFA in adipose tissue produces higher levels of inflammatory markers and this was accomplished by adipocyte death and reductions in glucose homeostasis and insulin sensitivity^64^.

In conclusion, our *in vitro* and *in vivo* data suggest that E2 regulates VEGFA via ESR1/HIF1A interaction in adipocytes and adipose tissue, which may lead to enhanced angiogenesis and improvements in adipose tissue function.

## Methods

### Cells 3T3-L1

Mouse 3T3-L1 preadipocytes were obtained from *American Type Culture Collection*, (ATCC® Number: CL-173TM), and their growth and differentiation were performed as previously described^64,65^. Briefly, two days after the cell´s confluency, they were induced to differentiation using DMEM high glucose, containing 10% FBS (FBS; Vitrocell Embriolife, Campinas, Brazil), 3-isobutyl- 1-methylxanthine (IBMX) 0.5 mM, dexamethasone (DEXA) 1μM and insulin 10 μg/ml (Sigma–Aldrich; St Louis, MO, USA). After differentiation, which lasted 10–12 days, fully-differentiated cells were ready to be used in the experiments. Twenty-four hours before the treatments, cells were exposed to DMEM without phenol-red and 10% of charcoal striped FBS (Sigma-Aldrich). Adipocytes were treated with different doses of E2 (0, 0.1, 10 and 100) nmol/L (nM) for 24 hours and/or agonist and antagonists of ESR1 and ESR2, followed by their collection and isolation for mRNA and protein analyses. The concentrations of E2, ESR1 and ESR2 agonists and antagonists were established according to previous reports^25,32,66–68^: we used 10 nM of the ESR1 agonist, PPT: 4,4′,4″-(4-Propyl-[1*H*]-pyrazole-1,3,5-triyl) *tris*phenol (Tocris, St. Louis, MO,USA,; ESR1 antagonist, MPP: 1,3-Bis(4-hydroxyphenyl)-4-methyl-5-[4-(piperidinylethoxy)phenol]-1H-pyrazol dihydrochloride (Tocris) (1µmol/L); ESR2 agonist, DPN: 2,3-bis(4-Hydroxyphenyl)-propionitrile) (Tocris) (100 nM); and 100 nM was used of the ESR2 antagonist: PHTPP, 4-[2-Phenyl-5,7-bis(trifluoromethyl) pyrazolo[1,5-a]pyrimidin-3-yl]phenol (Tocris).

### RNAi in cell culture

In this series of experiments, we used siRNA to knockdown *Esr1* in the presence and absence of estrogens to determine if *Esr1* mediates estrogens’ effects on *Vegfa*. To do this, 3T3-L1 cells were transfected with siRNA targeting murine *Esr1* (siGENOME Mouse siEsr1, GE Dharmacon, Lafayette, U.S.). As a control, cells were treated with an unrelated control siRNA (siGENOME non-targeting siRNA, GE Dharmacon), using Lipofectamine RNAi max transfection reagent (Thermo Fisher), according to the manufacturer’s instructions. 48h after siRNA transfection, cells were treated with estrogens and as indicated, and then lysed for RNA extraction.

### Animals

Female total body ESR1 knockout (ERKO) and wide type (WT) mice were purchased from Jackson Laboratories (C57BL Maine, USA, Stock No. 004744), and these mice are a functional knockout of ESR1; however, they do express a low level of a truncated form of ESR1 which is the result of alternate splicing of the transcript. Animals were group housed by genotype in a temperature controlled environment on a 12-h light/12-h dark illumination schedule and fed a standard pelleted diet with water provided ad libitum. Nine-month-old mice were used in the experiments. Samples from inguinal and gonadal white adipose tissues were collected and frozen immediately for Real time qPCR, or fixed in 4% paraformaldehyde overnight, then embedded in paraffin wax for immunofluorescence. All mouse experiments were carried out as approved by AAALCA (Association for Assessment and Accreditation for Laboratory Care) at the Pennington Biomed Center at LSU, and tissues were sent to Cedars-Sinai Medical Center (Los Angeles, USA) for processing. All methods were performed in accordance with the relevant guidelines and regulations.

### Real time qPCR

Cells or tissues were lysed in TRIzol® reagent (Thermo Fisher Scientific, Massachusetts, USA). RNA was extracted using phase separation reagent (BCP) (MRC®, Ohio, USA) and RNeasy kit (Qiagen®), according to the manufacturer´s instructions. The extracted RNA was measured by NanoDrop 2000c (Thermo Fisher Scientific). Total cDNA was obtained using the High Capacity cDNA Reverse Transcription kit (Thermo Fisher Scientific). Real-time qPCR was performed in duplicate by using Taqman Universal Mastermix II (Thermo Fisher Scientific). Taqman specific primers and QuantStudio 12K Flex Real-Time PCR System machine: Gapdh- Mm99999915_g1; Esr1- Mm00433149_m1; Esr2- Mm00599821_m1; Kdr- Mm01222421_m1; Ftl1- Mm00438980_m1; Tnf- Mm00443258_m1; Vegfa- 00437306_m1; Il6- Mm00446190_m1. Relative expression values were calculated from the threshold cycle (Ct) following the 2^−∆∆CT^ method, using *Gapdh* as the reference gene.

### Western blotting

Cells were homogenized in RIPA buffer. Protein concentration was determined by Bradford method (Bio-Rad Laboratories, CA). Proteins (25 μg) from cell lysates were separated by electrophoresis, and electrotransferred to nitrocellulose membrane Hybond-ECL (Amersham, Buckinghahmshire, UK). Later, membranes were stained with Ponceau S to be used as the loading control. Membranes were then blocked in TBS-T containing 5% non-fat powdered milk, followed by overnight incubation with primary antibodies (ESR1 Sc-542–1:1000; VEGFA Sc152–1:500; Santa Cruz, Dallas, TX, USA). Blot´s intensity was quantified by densitometry (ImageScanner III, GE Healthcare, Uppsala, Sweden). Results were expressed as arbitrary units in comparison to controls.

### Electrophoretic Mobility Shift Assay (EMSA)

Nuclear protein extraction was conducted according to (Xing *et al*.)^69^. Briefly, cells were washed with Phosphate buffered saline (PBS; NaCl 137 mM; KCL 2.68 mm, KH_2_PO_4_ 1.27 mm, Na_2_HPO_4_ 8.06 mM) and centrifuged 2000 *g* and 4 °C, for 5 min. The pellet was resuspended in lyses buffer (HEPES-KOH pH 7.9, 10 mM; MgCl_2_ 1.5 mM; KCl 10 mM; DTT 0.5 mM; PMSF 0.2 mM; leupeptin 5 µg/mL; aprotinin 15 µg/mL), incubated on ice for 10 min and 10% of Nonidet P-40 was added. After the centrifugation at 12000 *g* and 4 °C, for 20 min, the pellet was resuspended in 50 μl of extraction buffer (HEPES-KOH pH 7.9, 20 mM; MgCl_2_ 1.5 mM; EDTA 0.5 mM; DTT 0.2 mM; NaCl 420 mM; Glicerol 25%; PMSF 0.2 mM; leupeptin 5 µg/ml; aprotinin 15 µg/ml) and incubated on ice for 20 min.

To determine ER binding in the VEGFA gene, we used a wild-type double-stranded oligonucleotide containing the consensus region for the receptors in the 3′UTR of VEGFA gene 5′TCTACAAAAGCACCCCGCCCCTCTGG 3′^70^. Moreover, we used two mutated probes: 5′-TCTACAAttGCACCCaGCCCCTCTGG-3′; 5′-TCTACAAAAGCACCtCGCttCTCTGG-3′. To determine HIF1a binding in the VEGFA gene, we used the probe 5′-TGCATACGTGGGTTTCCACAG-3^53^.

Probes were end-labeled using T4Polynucleotide Kinase (Invitrogen Life Technologies, California, USA) and [γ-32P] ATP (PerkinElmer Life, Massachusetts, USA). The EMSA experiments were carried out according to those previously described^35^. The competitive binding assay was performed by adding unlabeled probes at 50-fold excess. To verify the presence of our protein of interest in the DNA-protein complex, specified antibodies were added in the final mixtures (Anti-HIF1A mouse monoclonal – Abcam 16066; Anti-ESR1 Rabbit polyclonal - Santa Cruz 542; Anti-ESR2 Goat polyclonal - Santa Cruz 6821 X; Supplementary Figures 2 and 3). The reaction mixture was then electrophoresed on a 4% nondenaturing polyacrylamide, after which gels were dried and exposed overnight to an X-ray film. The blots corresponded to each treatment were analyzed by scanner densitometry and the results of the binding activity were expressed as arbitrary units.

### Immunofluorescence

In this series, we verified protein expression and localization of VEGFA in adipose tissues from ERKO and WT mice. Adipose tissue was collected from different depots and embedded in paraffin wax and sectioned. The sections were incubated with primary antibody anti-VEGFA (1:100- sc-152) overnight at 4 °C in a moist chamber. Next, sections were incubated in a secondary antibody (Goat to rabbit- FITC, Abcam® ab6717) for 1h. Following which, sections were incubated with the membrane marker Wheat Germ Agglutinin (Life Technologies) for 30 min and Dapi (1:200, Abcam). Slides were mounted with Vectashield (Vector®, H1000). Images were acquired using Keyence BZ-9000 microscope, and analyzed by BZ-9000 analyzed software. Three sections from each mouse were stained, five pictures of each section were taken, and the fluorescence intensity was quantified using the ImageJ software (National Institutes of Health Bethesda, MD USA). The measurement of adipose cell surface area was done in the same pictures using the plugin Adiposoft (ImageJ software). Results were expressed as arbitrary units in comparison to controls.

### Data analysis

All data are presented as mean ± standard error of the mean (SEM). Data was tested for normality (*Levene Statistic*). Statistical significance between groups was determined by one-way ANOVA and *Bonferroni* as post-hoc-test. Two-tailed paired Student´s test was used as appropriated. P values < 0.05 were considered statistically significant. Statistical analyses and graphs were generated using GraphPad Prism 6 software (GraphPad software, San Diego, CA, USA).

## Electronic supplementary material

Supplementary Data 1
