# Supplementary material for: Estrogen receptor 1 (ESR1) regulates VEGFA in adipose tissue
Source: Sci Rep. 2017 Dec 1;7:16716. doi: 10.1038/s41598-017-16686-7 (PMC5711936; doi:10.1038/s41598-017-16686-7)
Supplement: Supplementary file 1 — Supplementary Data 1 [file 41598_2017_16686_MOESM1_ESM.doc]

**Supplementary data**

**Estrogen receptor 1 (ESR1) regulates VEGFA in adipose tissue**

Fatima LA^1*^, Campello RS^2^, de Souza Santos R^3^, Freitas HS^4^, Frank AP^5^, Machado UF^6#^, Clegg DJ^7#^.

**Corresponding-author:*

*^#^These authors jointly supervised study*

**
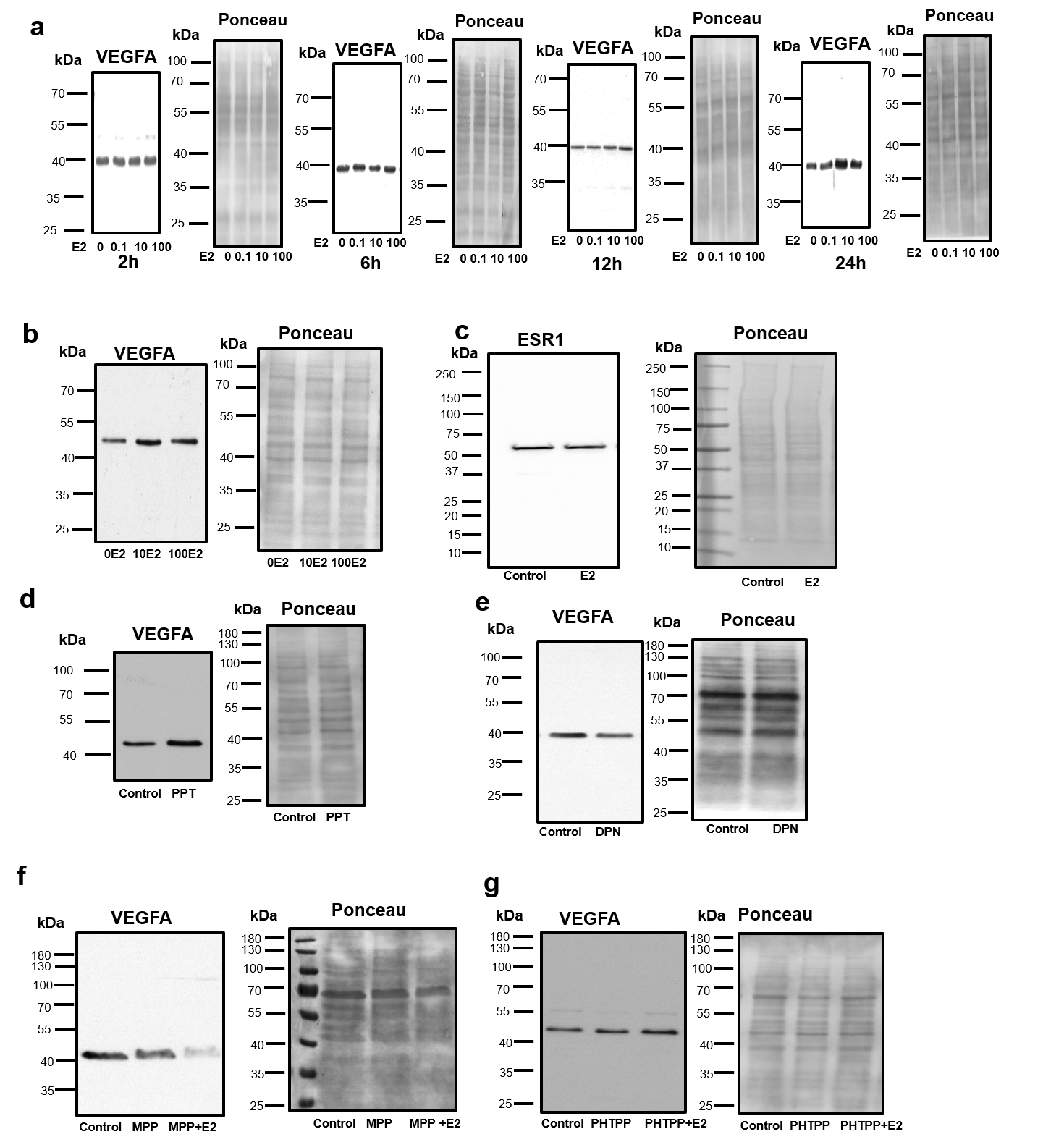
**

**Supplementary Figure 1:** Full-length blots of VEGFA and ESR1 shown as cropped images in figures: 1 (a); 2 (b); 3 (c and d) and 4 (e and f).

**
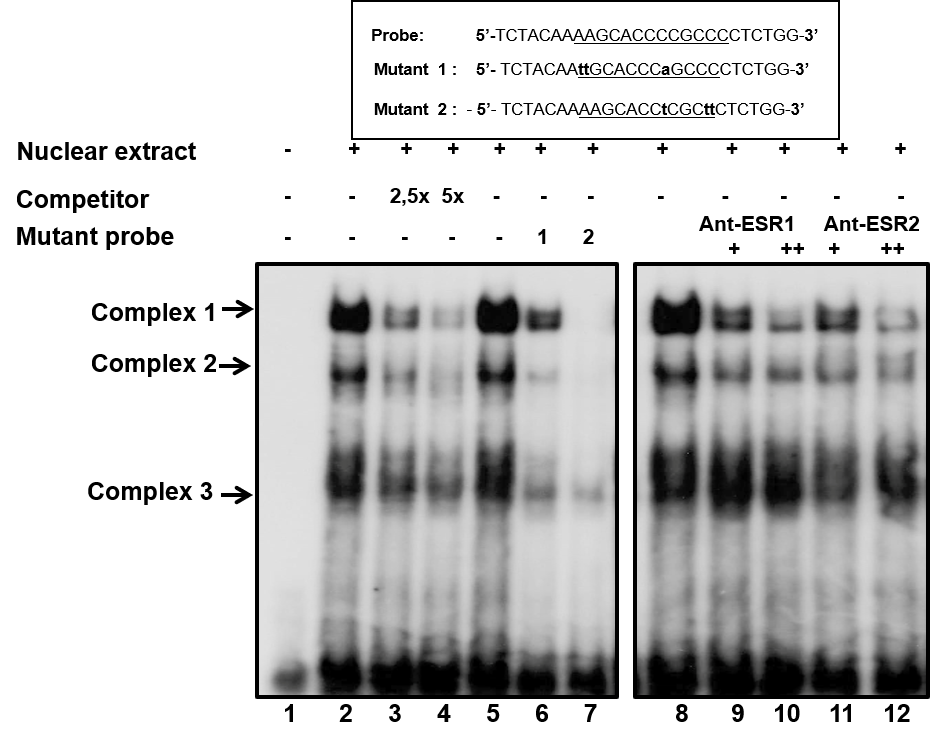
**

**Supplementary Figure 2:** Identification of nuclear proteins interacting with the ER site of the mouse *Vegfa* promoter in 3T3-L1 cells. P4-labeled ERE oligonucleotide was incubating in the absence (-) in lane 1 or presence (+), in other lanes, of 3T3-L1 adipocytes nuclear extract. For competition analysis, excess amount of non-labeled competitor was added (2.5- and 5-fold molar) lanes 3 and 4. Two muted probes (lanes 6 and 7) containing a 3-bp substitution in the VEGFA binding were used to verify the specificity and stability of the complexes. Antibodies against ESR1 (lanes 9 and 10) and ESR2 (lanes 11 and 12) in two different concentrations (+=2µg and ++=4 2µg) were used to determine the ER subtypes in the composition of the complexes. Complexes 1 and 2 were clearly modulated by addition of anti-ESR1 and anti-ESR2 antibodies, indicating the presence of both receptors in these complexes.


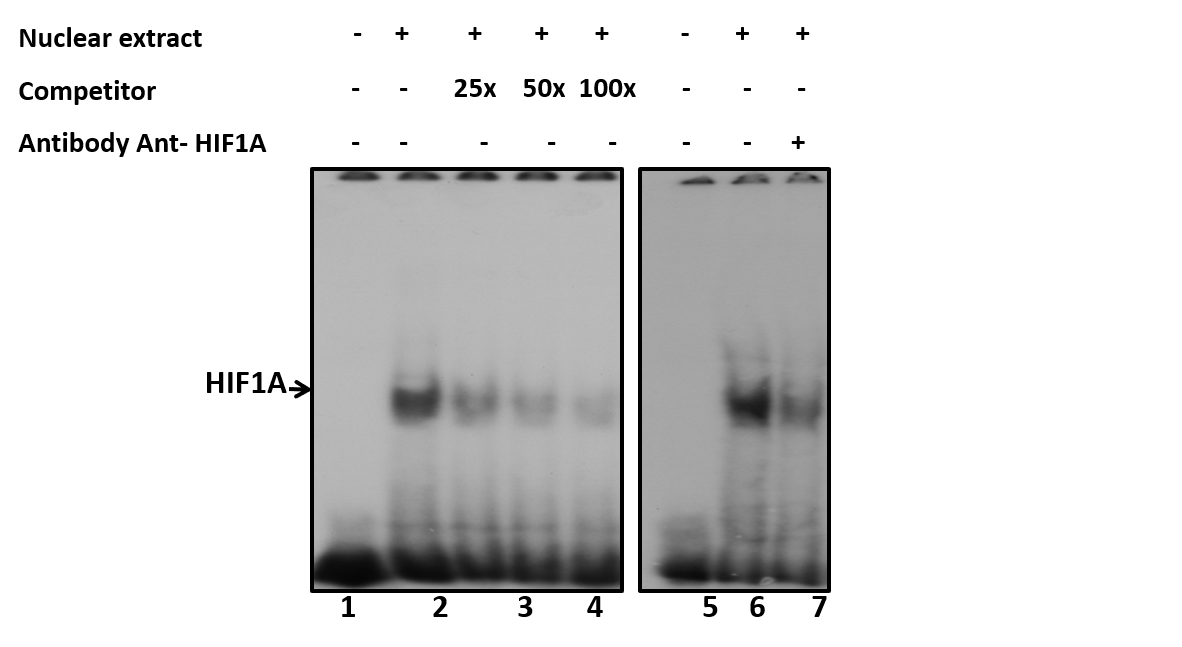


**Supplementary Figure 3:** Identification of nuclear proteins interacting with the HIF1A site of the mouse *Vegfa* promoter in 3T3-L1 cells. P4-labeled ERE oligonucleotide was incubated in the absence (-) in lane 1 or presence (+) of 4 µg of 3T3-L1 adipocytes nuclear extract in other lanes. For the competition analysis, excess amount of non-labeled competitor was added (25-, 50- and 100-fold molar) and represented in lanes 3, 4 and 5. Antibody against HIF1A (lane 7) was used to determine the presence of HIF1A in the DNA/protein complex.


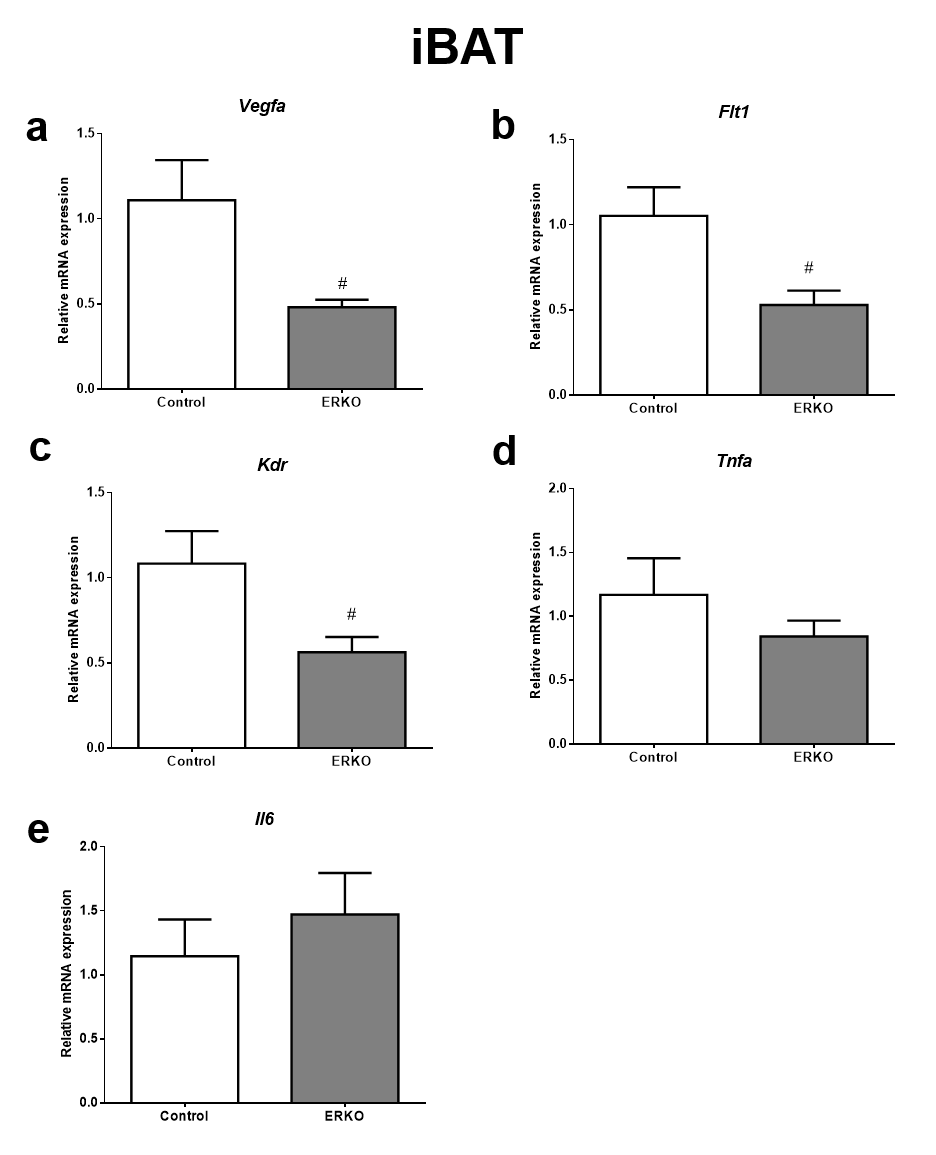


**Supplementary Figure 4**: Relative mRNA expression of *Vegfa, VEGFA* receptors, and inflammatory markers in iBAT from WT and ERKO, female mice. A: *Vegfa;* B*: Flt;* C*: Kdr;* D*: Tnfa* and E: *Il6*. Data on the graphs are means ± SEM of 4-7 samples, # p< 0.05 versus WT.
